# Work volume is an important variable in determining the degree of inhibitory control improvements following resistance exercise

**DOI:** 10.14814/phy2.14527

**Published:** 2020-08-09

**Authors:** Keigo Tomoo, Tadashi Suga, Takeshi Sugimoto, Daichi Tanaka, Kento Shimoho, Kento Dora, Ernest Mok, Shawn Matsumoto, Hayato Tsukamoto, Shingo Takada, Takeshi Hashimoto, Tadao Isaka

**Affiliations:** ^1^ Faculty of Sport and Health Science Ritsumeikan University Kusatsu Shiga Japan; ^2^ Department of Neuroscience University of Texas at Austin Austin TX USA; ^3^ Department of Sports Education Faculty of Lifelong Sport Hokusho University Ebetsu Hokkaido Japan

**Keywords:** brain health, cognitive function, exercise adherence, lactate

## Abstract

We previously determined that improvement in cognitive inhibitory control (IC) immediately after localized resistance exercise was greater for high‐intensity resistance exercise (HRE) than for low‐intensity resistance exercise (LRE). However, our previous study used the same total repetitions (i.e., same repetitions per set) between HRE and LRE; therefore, the difference in postexercise IC improvement might be due to a difference in work volume (i.e., intensity × total repetitions). In this study, we compared the effect of high‐volume (HV)‐LRE to that of volume‐matched HRE on postexercise IC improvements. Twenty‐two healthy, young males performed both HV‐LRE and HRE in a crossover design. Exercise loads for HV‐LRE and HRE were set at 35% and 70% of one‐repetition maximum, respectively. The bilateral knee extension exercises for HV‐LRE and HRE were programmed for six sets with 20 and 10 repetitions, respectively, per set. IC was measured using the color‐word Stroop task (CWST) at six time points; baseline, pre‐exercise, immediate postexercise, and every 10 min during the 30‐min postexercise recovery period. The reverse‐Stroop interference score decreased significantly immediately after HV‐LRE and HRE compared with that before each exercise (decreasing rate >34 and >38%, respectively, vs. baseline and pre‐exercise; all *p*s < .05), and the decreased score remained significant until 20 min after both protocols (decreasing rate >40 and >38%, respectively, vs. baseline and pre‐exercise; all *p*s < .05). The degree of the postexercise IC improvements did not differ significantly between the two protocols. These findings suggest that HV‐LRE improves IC in a similar manner to volume‐matched HRE.

## Graphical abstract

An acute bout of high‐intensity resistance exercise (HRE) remarkably improves cognitive inhibitory control (IC). This study compared the effect of high‐volume low‐intensity resistance exercise (HV‐LRE) and volume‐matched HRE on postexercise IC improvements. Despite the use of a lower exercise load, the degree of IC improvements following HV‐LRE was comparable to that following HRE.

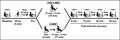

## 1 INTRODUCTION

Skeletal muscle weakness, as seen in decreased muscle mass and strength, is a prominent factor that indicates poor prognosis in older individuals and patients with chronic diseases (Ruiz et al., 2008). Many people with skeletal muscle weakness also present decreased cognitive function (Chang, Hsu, Wu, Huang, & Han, 2016), which is also a poor prognostic factor (DeCarli, 2003). Because a complication of skeletal muscle weakness and decreased cognitive functions additively exacerbate physical inactivity (Atkinson et al., 2005), resolution of this public health problem worldwide is now urgent. Resistance exercise is the most beneficial strategy for increasing and/or maintaining skeletal muscle mass and strength (American College of Sports Medicine, 2009; Williams et al., 2007). Furthermore, recent studies determined that long‐term resistance exercise could improve cognitive functions in various populations, including older individuals and patients with chronic diseases (Northey, Cherbuin, Pumpa, Smee, & Rattray, 2018; Wilke et al., 2019). Therefore, resistance exercise is recognized as the effective strategy in preventing both skeletal muscle weakness and decreased cognitive function.

Cognitive inhibitory control (IC) is defined as the suppression of behavior in response to either internal or external stimuli (Ozonoff & Strayer, 1997), which is necessary to prevent the implementation of an unrequired action (Coxon, Stinear, & Byblow, 2007). Previous studies reported that whole‐body resistance exercise involving multiple events (e.g., combinations of leg press, leg curl, bench press, and lat pull‐down, etc.) could improve IC immediately after exercise in some populations (Alves et al., 2012; Brush, Olson, Ehmann, Osovsky, & Alderman, 2016; Chang, Tsai, Huang, Wang, & Chu, 2014; Engeroff, Niederer, Vogt, & Banzer, 2019; Johnson et al., 2016). Additionally, we demonstrated an immediate positive effect of localized resistance exercise, performed with knee extension, on postexercise IC (Tsukamoto, Takenaka, et al., 2017). However, little is known about the sustainable effect (i.e., duration of postexercise IC improvements) of resistance exercise‐induced improvements in cognitive functions, including IC, during postexercise recovery. Brush et al. (2016) reported that although IC improved 15 min after whole‐body resistance exercise with high‐intensity load, this IC improvement reversed during the 180‐min postexercise recovery. Johnson et al. (2016) reported that although IC improved immediately after moderate‐intensity whole‐body resistance exercises with a 10‐ or 30‐min duration, this IC improvement reversed during the 30‐min postexercise recovery. Considering the results of these previous studies, the extent of postexercise IC improvements that can be sustained after resistance exercise remains unknown. We previously examined the effect of aerobic exercise protocols on IC improvement dynamics throughout the 30‐min postexercise recovery period and determined that this sustainable effect is different among the aerobic exercise protocols (Hashimoto et al., 2018; Tanaka et al., 2018; Tsukamoto et al., 2016a, 2016b, 2018; Tsukamoto, Takenaka, et al., 2017). Therefore, in addition to the immediate effect, the sustainable effect is an important indicator in understanding the potential effect of aerobic exercise protocols on IC. Taken together, the changes in IC improvements during the postexercise recovery periods may be useful information for creating an effective resistance exercise program to improve IC.

Our previous study determined that the immediate postexercise improvement in IC was greater following high‐intensity resistance exercise (HRE) than following low‐intensity resistance exercise (LRE; Tsukamoto, Suga, et al., 2017). However, we used the same total repetitions (i.e., same repetitions per set) between the two protocols. In the case of aerobic exercise, exercise volume, which is the product of exercise intensity and duration, plays an important role in determining the degree of exercise‐induced IC improvements (Tsukamoto, Takenaka, et al., 2017). Hence, the difference in immediate postexercise IC improvement between LRE and HRE may be due to the difference in work volume, which is the product of exercise load and total repetitions. Considering this, we hypothesized that even with lower loads, IC improvements following high‐volume (HV)‐LRE with increased total repetitions may approach that following HRE. Therefore, the primary purpose of the present study was to compare the effect of HV‐LRE and volume‐matched HRE on postexercise IC improvements. Additionally, the secondary purpose of the present study was to determine the sustainable effect of IC improvements during the 30‐min postexercise recovery in HV‐LRE and HRE.

## 2 METHODS

### 2.1 Subjects

Twenty‐two healthy, young males (age: 21.2 ± 0.5 years, body height: 174.1 ± 0.9 cm, body weight: 66.2 ± 1.2 kg) participated in this study. Prior to this study, we calculated the required sample size utilizing an effect size of 0.31, an α‐level of 0.05, and a β‐level of 0.2 (80% power), based on the data of a two‐way repeated‐measures analysis of variance for main outcome measure (i.e., the reverse‐Stroop interference score) in our previous study (Tsukamoto et al., 2016b). The calculated necessary number of subjects was 14; thus, this study has an adequate sample size to ensure statistical power and sensitivity. The subjects were recreationally active and participated in physical exercise (e.g., resistance exercise and/or aerobic exercise) for 2–4 hr per week. The subjects provided written informed consent upon having the experimental procedures and potential risks described to them. The color‐word Stroop test (CWST) was performed using the subject's right hand. All subjects were considered right‐hand dominant, which was ascertained by asking each subject which hand they preferred to use for writing. Furthermore, they were free of any known neurological, cardiovascular, and pulmonary disorders, as well as free from color‐blindness and abnormal vision. All subjects were instructed to avoid strenuous physical activity in the 24 hr prior to each experimental session. Each subject also abstained from food, caffeine, and alcohol for 12 hr prior to each experiment, and was not taking any medications that may affect cognitive performance. This study was approved by the Ethics Committee of Ritsumeikan University and conducted according to the Declaration of Helsinki.

### 2.2 Experimental design

This study conducted a crossover design experiment in which subjects were required to attend the laboratory for a total of three separate occasions. In the first visit, the subjects completed a familiarization visit where they practiced the three types of CWST for each a minimum of 10 times until they achieved consistent scores. During this visit, one‐repetition maximum (1‐RM) of the bilateral knee extension was also measured for the main experimental equipment.

**FIGURE 1:**
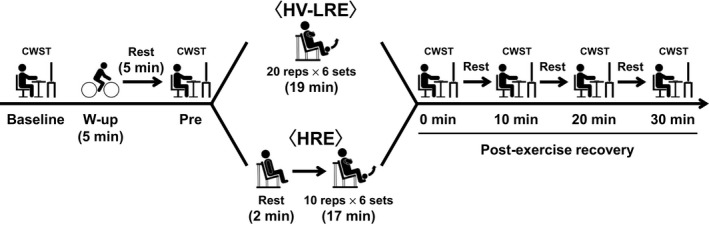
Experimental procedures of high‐volume low‐intensity resistance exercise (HV‐LRE) and high‐intensity resistance exercise (HRE) sessions. Exercise loads for the HV‐LRE and HRE were set at 35% and 70% of one‐repetition maximum, respectively. The bilateral knee extension exercise sessions for the HV‐LRE and HRE were programmed for six sets with 20 and 10 repetitions, respectively, per set. The color‐word Stroop task (CWST)‐measured IC was administered at baseline, pre‐exercise (i.e., Pre), immediately after exercise (i.e., 0 min at postexercise recovery), and every 10 min during the 30‐min postexercise recovery period

Experimental procedures of HV‐LRE and HRE are presented in Figure 1. In the second and third visits, subjects abstained from food, caffeine, and alcohol for 12 hr prior to the experiment, and were not taking any medications that may affect cognitive performance. On each experiment, the subjects practiced the three CWST types for each a minimum of five times before the experimental session to minimize the learning effect. Then, the subjects rested for 5 min before undergoing physiological and psychological parameter measurements. After these baseline data were collected, the subjects performed the baseline CWST.

Next, the subjects performed a warm‐up exercise at 50 W for 5 min using a bicycle ergometer (Life Fitness), similar to our previous study (Tsukamoto, Suga, et al., 2017). Heart rate (HR) and rating of perceived exertion (RPE) at the end of the warm‐up exercise were recorded. After the warm‐up exercise, the subjects rested for 5 min and then performed the pre‐exercise CWST to confirm if the warm‐up exercise did not affect IC.

Subsequently, the subjects completed either HV‐LRE or HRE. The CWST was performed again immediately after the completion of the exercise session and then repeated three times at 10‐min intervals during the 30‐min postexercise recovery period in order to evaluate the sustainable effects of postexercise IC improvements, as in our previous studies (Hashimoto et al., 2018; Tanaka et al., 2018; Tsukamoto et al., 2016a, 2016b, 2018; Tsukamoto, Takenaka, et al., 2017).

HR, RPE, and quadriceps femoris EMG activity during exercise session were measured in every set to determine cardiovascular, and perceptual, and neuromuscular responses. Capillary blood samples were taken from the fingertip and collected immediately before all six CWSTs to assess the effect of metabolic conditions on IC. Felt arousal scale (FAS) and visual analogue scale (VAS) were measured immediately after all CWSTs to assess the effect of psychological conditions on IC.

### 2.3 Experimental protocols

All subjects performed both HV‐LRE and HRE in a randomized and counterbalanced order. Our previous study used 40% and 80% of 1‐RM for LRE and HRE, respectively (Tsukamoto, Suga, et al., 2017). However, some subjects needed to lower their exercise loads to maintain proper form for the knee extension during HRE. The present study required the use of the same work volume between HV‐LRE and HRE. Hence, the HV‐LRE and HRE were set at 35% and 70% of 1‐RM, respectively, and all subjects fully completed each exercise session. Both protocols were performed with the bilateral knee extension using an exercise machine (Life Fitness). To perform the same exercise volume, the bilateral knee extension exercises of HV‐LRE and HRE were programmed for six sets with 20 and 10 repetitions (1‐s concentric contraction/1‐s eccentric contraction), respectively, per set. The concentric and eccentric contraction paces were assisted with an auditory metronome. Rest intervals between sets for both protocols lasted 3 min. The two conditions were separated by at least 72 hr, and the maximum interval between the conditions was 7 days.

### 2.4 1‐RM

On the first visit, the subjects’ 1‐RM was determined by a successful concentric contraction of bilateral knee extension to calculate exercise loads for both HV‐LRE and HRE, as previously described (Suga et al., 2009; Takada et al., 2012; Tsukamoto, Suga, et al., 2017). The mean value of the bilateral knee extension 1‐RM in all subjects was 116 ± 4 kg. The mean values of loads for HV‐LRE and HRE were 41 ± 1 and 81 ± 3 kg, respectively.

### 2.5 HR

HR was measured continuously via telemetry (RS400; Polar Electro Japan). Peak HR was collected for every set during the exercise session, and the mean value of all six sets was calculated for analysis.

### 2.6 RPE

Borg's RPE scale was measured to assess perceived exertion expended during cycling warm‐up and resistance exercise, and this scale ranged from 6 (no exertion) to 20 (maximal exertion) (Borg, 1982). RPE was collected for every set during the exercise session, and the mean value of all six sets was calculated for analysis.

### 2.7 Quadriceps femoris EMG activity

Prior to the application of electrodes, the subject's skin was shaved, abraded, and cleaned with alcohol to minimize skin impedance. EMG signals were recorded with surface electrodes from the quadriceps vastus lateralis, vastus medialis, and rectus femoris muscles. The EMG signals were amplified 1,000 times, band‐pass filtered between 10 and 500 Hz, and sampled at 1,000 Hz (MQ‐Air; Kissei Comtech). The EMG activity of each muscle during HV‐LRE and HRE was quantified as the integration of the rectified EMG (iEMG) over 1 s. The iEMG was normalized to the highest EMG (the average value over 1 s) that was obtained during the two trials of knee extension maximal voluntary contractions, which was measured after CWST was completed at the 30‐min postexercise recovery period. Peak iEMG activities of the three quadriceps femoris muscles were calculated for every set during the experimental session. The peak iEMGs of each muscle were averaged on the last 10 repetitions for HV‐LRE and all the 10 repetitions for HRE were averaged in their respective categories. Mean values of peak iEMGs of all six sets for each muscle were calculated for analysis.

### 2.8 Blood metabolites

Blood glucose and lactate levels were measured using a glucose (Medisafe FIT Blood Glucose Meter; Terumo) and lactate analyzer (Lactate Pro 2; Arkray), respectively.

### 2.9 Psychological conditions

FAS is a 6‐point, single‐item scale ranging from 1 (low‐arousal) to 6 (high arousal) (Svebak & Murgatroyd, 1985). VAS consisted of questions of three psychological types that assess mental fatigue, concentration, and motivation. Each VAS was labeled from 0 (i.e., not at all) to 100 mm (i.e., extremely). Subjects drew lines to indicate their response.

### 2.10 CWST‐measured IC

The CWST was administered to determine IC (Stroop, 1935), as previously described (Hashimoto et al., 2018; Tanaka et al., 2018; Tsukamoto et al., 2018; Tsukamoto, Suga, et al., 2017; Tsukamoto et al., 2016a, 2016b; Tsukamoto, Takenaka, et al., 2017). In brief, the stimulus words were four color names (“RED,” “YELLOW,” “GREEN,” and “BLUE”), and they were presented on a 98‐inch display. The three types of the CWST consisted of two color text tasks (i.e., congruent and incongruent tasks) and one control black text task (i.e., neutral task). One trial of each type of the CWST consisted of 24 stimulus words. The three CWST types were repeated for each three trials. The reaction time and response accuracy for each trial were collected and the mean values of the three trials of each CWST type were calculated for analysis. The IC was assessed using the reverse‐Stroop interference score, which was defined as the difference between reaction times of the neutral and incongruent tasks. The reverse‐Stroop interference score was calculated as [(reaction time of incongruent task − reaction time of neutral task)/reaction time of neutral task × 100] (Ikeda, Hirata, Okuzumi, & Kokubun, 2010).

### 2.11 Statistical analysis

Data are expressed as the mean ± *SEM*. Mean values of measured variables (i.e., HR, RPE, and quadriceps femoris EMG activity) during HV‐LRE and HRE were compared using a paired Student's *t* test. Changes in measured variables throughout the two experimental sessions were analyzed using two‐way (2 conditions × 6 times) repeated‐measures analysis of variance with post hoc Bonferroni correction. If the sphericity assumption was not met, Greenhouse‐Geisser corrections were used. Specific difference between the two protocols was identified with a paired Student's *t* test. Statistical significance level was defined at *p* < .05. All statistical analyses were conducted using IBM SPSS software (Ver. 19.0, IBM Corp).

The Cohen's *d* effect size using the pooled standard deviation was calculated to determine the magnitude of a difference in measured variables between condition or time points (Cohen, 1992). The magnitude of Cohen's *d* effect size was interpreted as small (0.20–0.49), medium (0.50–0.79), and large (>0.80). Partial eta squared ($\eta_{p}^{2}$) values were determined as a measure of the effect size for main effects of condition and time and an interaction effect.

## 3 RESULTS

### 3.1 Measured variables during warm‐up and exercise sessions

The HR (88.6 ± 2.0 and 90.5 ± 2.1 bpm) and RPE (8.3 ± 0.4 and 8.5 ± 0.3) at the end of warm‐up exercise did not differ significantly between HV‐LRE and HRE sessions (both *p*s > .05).

Mean values of HR, RPE, and quadriceps femoris EMG activity during HV‐LRE and HRE are shown in Table 1. Mean values of HR and RPE did not differ significantly between HV‐LRE and HRE. In contrast, mean values from all three quadriceps femoris EMG activities were significantly higher during HRE than during HV‐LRE (all *p*s < .01; *d*s = 0.68–1.24).

**TABLE 1:** Mean values of heart rate, rating of perceived exertion, and quadriceps femoris electromyographic activity during high‐volume low‐intensity resistance exercise (HV‐LRE) and high‐intensity resistance exercise (HRE)

	HV‐LRE	HRE
Heart rate, bpm	122.3 ± 3.5	122.0 ± 3.4
Rating of perceived exertion	15.2 ± 0.3	15.1 ± 0.2
Peak EMG		
Vastus lateralis, % of MVC	80.6 ± 4.3^a^	101.1 ± 6.2
Vastus medialis, % of MVC	79.3 ± 5.3^a^	98.2 ± 6.5
Rectus femoris, % of MVC	65.1 ± 3.6^a^	103.0 ± 8.5

Note

Values are presented as Mean ± *SEM*. Mean value of all six sets for each variable was calculated for analysis.

Abbreviations: EMG, electromyography; MVC, maximal voluntary contraction.

^a^ Significant difference (*p* < .01) between conditions.

### 3.2 Changes in blood metabolites throughout experimental session

Changes in blood glucose and lactate levels throughout HV‐LRE and HRE sessions are shown in Table 2. Blood glucose analysis revealed a significant main effect for time (*F*_(2.76, 57.88)_ = 4.59, *p* = .007, $\eta_{p}^{2}=0.18$). Post hoc comparisons for the time main effect indicated trends against significance for lower blood glucose at 20 min after exercise than that before exercise (*p* = .60 and .57 vs. baseline and pre‐exercise, respectively). Blood lactate analysis revealed significant main effects for condition (*F*_(1,21)_ = 5.89, *p* = .024, $\eta_{p}^{2}=0.22$) and time (*F*_(1.41, 29.54)_ = 186.67, *p* < .001, $\eta_{p}^{2}=0.90$). A trend against significance was also obtained for an interaction effect (*F*_(2.23, 46.90)_ = 2.65, *p* = .075, $\eta_{p}^{2}=0.11$). Post hoc comparisons for the condition main effect indicated that blood lactate was significantly higher for HV‐LRE than for HRE (*p* = .024). Post hoc comparisons for the time main effect indicated that blood lactate was significantly higher immediately after exercise than before exercise (both *p*s < .001 vs. baseline and pre‐exercise). The higher blood lactate remained significant until the 30‐min postexercise recovery period (all *p*s < .001 vs. baseline and pre‐exercise). Blood lactate levels from 10 to 30 min after exercise were significantly higher with HV‐LRE than with HRE (all *p*s < .05, *d*s = 0.35–0.39). A trend against such significance was observed for blood lactate immediately after exercise (*p* = .081, *d* = 0.031).

**TABLE 2:** Changes in blood metabolites throughout HV‐LRE and HRE sessions

	Baseline	Pre‐EX	Post‐EX 0 min	Post‐EX 10 min	Post‐EX 20 min	Post‐EX 30 min
Blood glucose (mg/dl)						
HV‐LRE	93.8 ± 1.3	94.1 ± 1.2	90.5 ± 1.3	90.0 ± 1.3	89.3 ± 1.0	91.1 ± 1.0
HRE	93.7 ± 1.4	92.6 ± 1.0	92.2 ± 1.4	92.2 ± 1.4	90.8 ± 1.3	91.4 ± 1.1
Blood lactate (mM)^*^						
HV‐LRE	1.0 ± 0.0	1.1 ± 0.1	8.8 ± 0.5	6.0 ± 0.4*	4.1 ± 0.4*	3.0 ± 0.3*
HRE	1.0 ± 0.0	1.0 ± 0.1	8.0 ± 0.5	5.2 ± 0.4	3.5 ± 0.3	2.5 ± 0.2

Note

Values are presented as Mean ± *SEM*.

Abbreviations: Post‐EX 0, immediately after exercise; Post‐EX 10, 10‐min post‐exercise recovery period; Post‐EX 20, 20‐min post‐exercise recovery period; Post‐EX 30, 30‐min post‐exercise recovery period; Pre, before exercise.

^a^ Significant difference (*p* < .001) between pre‐exercise periods (i.e., Baseline and Pre‐EX) and Post‐EX 0 min.

^b^ Significant difference (*p* < .001) between pre‐exercise periods and Post‐EX 10 min.

^c^ Significant difference (*p* < .001) between pre‐exercise periods and Post‐EX 20 min.

^d^ Significant difference (*p* < .001) between pre‐exercise periods and Post‐EX 30 min.

^e^ Significant difference (*p* < .001) between Post‐EX 0 min and Post‐EX 10 min.

^f^ Significant difference (*p* < .001) between Post‐EX 0 min and Post‐EX 20 min.

^g^ Significant difference (*p* < .001) between Post‐EX 0 min and Post‐EX 30 min.

^h^ Significant difference (*p* < .001) between Post‐EX 10 min and Post‐EX 20 min.

^i^ Significant difference (*p* < .001) between Post‐EX 10 min and Post‐EX 30 min.

^j^ Significant difference (*p* < .001) between Post‐EX 20 min and Post‐EX 30 min.

* Significant difference (*p* < .05) between conditions.

### 3.3 Changes in the CWST‐measured IC throughout experimental session

Changes in reaction times on three types of the CWST throughout HV‐LRE and HRE sessions are shown in Table 3. Reaction time analyses for all three CWST types revealed a significant main effect for time (*F*_(5,105)_ = 13.03, *p* < .001, $\eta_{p}^{2}=0.38$ for congruent task; *F*_(5,105)_ = 9.07, *p* < .001, $\eta_{p}^{2}=0.30$ for neutral task; *F*_(3.23, 67.83)_ = 19.77, *p* < .001, $\eta_{p}^{2}=0.49$ for incongruent task). Post hoc comparisons for the time main effect of congruent task indicated that reaction time was significantly shorter immediately after exercise than before exercise (both *p*s < .001 vs. baseline and pre‐exercise). The shorter congruent reaction time remained significant until the 20‐min postexercise recovery period (all *p*s = .05). Post hoc comparisons for the time main effect of neutral task indicated that reaction time was significantly shorter immediately after exercise than before exercise (*p* = .002 and .001 vs. baseline and pre‐exercise, respectively). The shorter neutral reaction time remained significant until the 10‐min postexercise recovery period (*p* = .007 and .036 vs. baseline and pre‐exercise, respectively). Post hoc comparisons for the time main effect of incongruent task indicated that reaction time was significantly shorter immediately after exercise than before exercise (both *p*s < .001 vs. baseline and pre‐exercise). The shorter incongruent reaction time remained significant until the 10‐min postexercise recovery period (all *p*s < .05 vs. baseline and pre‐exercise). Trends against significance for shorter incongruent reaction time at 30 min after exercise than that before exercise were also obtained (*p* = .056 and .055 vs. baseline and pre‐exercise, respectively). Response accuracy analyses for all three CWST types revealed no significant main effects for time and condition or no significant interaction effect (data not shown).

**TABLE 3:** Changes in reaction times of the three color‐word Stroop tasks throughout HV‐LRE and HRE sessions

	Baseline	Pre‐EX	Post‐EX 0 min	Post‐EX 10 min	Post‐EX 20 min	Post‐EX 30 min
Congruent task (msec)^a^, ^b^, ^c^, ^e^, ^g^						
HV‐LRE	9,364 ± 315	9,321 ± 327	8,700 ± 294	8,637 ± 251	8,905 ± 311	9,030 ± 265
HRE	9,352 ± 331	9,336 ± 343	8,649 ± 282	8,892 ± 317	8,916 ± 318	9,034 ± 329
Neutral task (msec)^a^, ^b^, ^d^, ^e^						
HV‐LRE	9,687 ± 305	9,548 ± 300	9,151 ± 271	9,343 ± 290	9,410 ± 294	9,470 ± 310
HRE	9,713 ± 343	9,633 ± 318	9,226 ± 314	9,385 ± 334	9,496 ± 334	9,646 ± 331
Incongruent task (msec)^a^, ^b^, ^c^, ^e^, ^f^, ^g^						
HV‐LRE	10,614 ± 349	10,453 ± 332	9,721 ± 299	9,823 ± 302	9,943 ± 314	10,076 ± 343
HRE	10,656 ± 377	10,558 ± 356	9,773 ± 337	9,901 ± 355	10,063 ± 362	10,273 ± 363

Note

Values are presented as Mean ± *SEM*.

^a^ Significant difference (*p* < .05) between pre‐exercise periods and Post‐EX 0 min.

^b^ Significant difference (*p* < .05) between pre‐exercise periods and Post‐EX 10 min.

^c^ Significant difference (*p* < .001) between pre‐exercise periods and Post‐EX 20 min.

^d^ Significant difference (*p* < .001) between Post‐EX 0 min and Post‐EX 20 min.

^e^ Significant difference (*p* < .001) between Post‐EX 0 min and Post‐EX 30 min.

^f^ Significant difference (*p* < .001) between Post‐EX 10 min and Post‐EX 20 min.

^g^ Significant difference (*p* < .001) between Post‐EX 10 min and Post‐EX 30 min.

**FIGURE 2:**
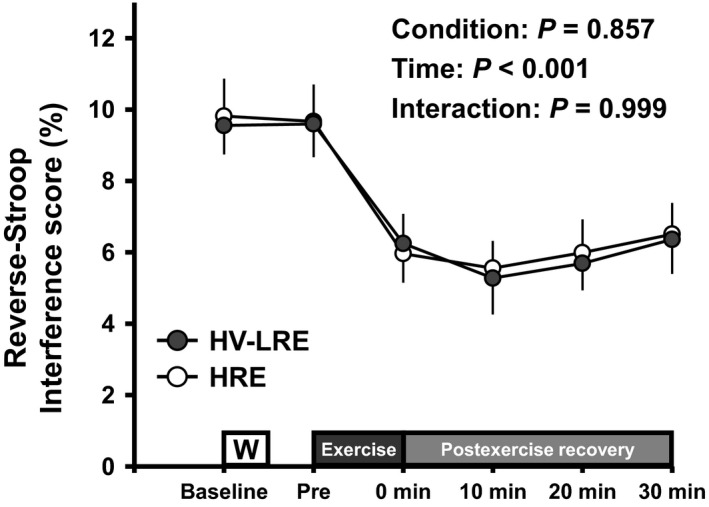
Changes in the reverse‐Stroop interference scores throughout HV‐LRE and HRE sessions. Values are presented as Mean ± *SEM*. *Significant difference (*p* < .05) from Baseline and Pre

Changes in the reverse‐Stroop interference scores throughout HV‐LRE and HRE sessions are presented in Figure 2. The reverse‐Stroop interference score (i.e., IC) analysis revealed a significant main effect for time (*F*_(5,105)_ = 11.70, *p* < .001, $\eta_{p}^{2}=0.36$). Post hoc comparisons for the time main effect indicated that the reverse‐Stroop interference score was significantly lower immediately after exercise than before exercise (*p* = .001 and .002 vs. baseline and pre‐exercise, respectively). The lower score remained significant until the 30‐min postexercise recovery period (all *p*s < .05 vs. baseline and pre‐exercise).

### 3.4 Changes in psychological conditions for the CWST throughout experimental session

Changes in psychological parameters for the CWST throughout HV‐LRE and HRE sessions are shown in Table 4. Arousal analysis revealed a significant main effect for time (*F*_(2.53, 53.09)_ = 30.58, *p* < .001, $\eta_{p}^{2}=0.59$). Post hoc comparisons for the time main effect indicated that arousal was significantly higher immediately after exercise than before exercise (both *p*s < .001 vs. baseline and pre‐exercise). The higher arousal remained significant until the 30‐min postexercise recovery period (all *p*s < 0.05 vs. baseline and/or pre‐exercise). Analyses of all three VAS‐measured psychological conditions revealed significant effects for time (*F*_(2.36, 49.45)_ = 28.67, *p* < .001, $\eta_{p}^{2}=0.58$ for mental fatigue; *F*_(2.56, 54.55)_ = 5.11, *p* = .005, $\eta_{p}^{2}=0.12$ for ability to concentrate; *F*_(2.32, 48.85)_ = 5.53, *p* = .005, $\eta_{p}^{2}=0.21$ for motivation). With regard to post hoc comparisons for these time main effects, mental fatigue was significantly higher immediately after exercise than before exercise (both *p*s < .001 vs. baseline and pre‐exercise). The higher mental fatigue remained significant until the 30‐min postexercise recovery period (all *p*s < .001 vs. baseline and/or pre‐exercise). Concentration and motivation were significantly higher immediately after exercise than before exercise (all *p*s < .05 vs. baseline and pre‐exercise).

**TABLE 4:** Changes in psychological conditions for the color‐word Stroop task throughout HV‐LRE and HRE sessions

	Baseline	Pre‐EX	Post‐EX 0 min	Post‐EX 10 min	Post‐EX 20 min	Post‐EX 30 min
Arousal^a^, ^b^, ^c^, ^e^, ^f^, ^g^, ^h^, ^i^						
HV‐LRE	2.3 ± 0.1	2.8 ± 0.1	4.6 ± 0.2	3.7 ± 0.2	3.2 ± 0.2	3.2 ± 0.2
HRE	2.5 ± 0.1	2.8 ± 0.1	4.7 ± 0.2	3.9 ± 0.2	3.3 ± 0.2	3.3 ± 0.2
Mental fatigue (mm)^a^, ^b^, ^c^, ^d^, ^f^, ^g^, ^h^, ^i^						
HV‐LRE	18.7 ± 4.5	21.5 ± 4.5	60.3 ± 5.0	52.2 ± 5.2	45.6 ± 5.6	36.9 ± 5.8
HRE	19.1 ± 3.4	21.1 ± 4.1	62.1 ± 4.9	51.6 ± 5.4	39.7 ± 5.2	40.0 ± 5.8
Concentration (mm)^a^						
HV‐LRE	58.4 ± 5.5	58.7 ± 5.2	76.6 ± 3.0	70.4 ± 2.6	61.3 ± 5.0	64.5 ± 5.5
HRE	58.4 ± 4.9	61.3 ± 3.8	74.0 ± 4.1	63.2 ± 3.6	63.4 ± 4.3	61.2 ± 4.3
Motivation (mm)^a^, ^e^, ^f^, ^g^						
HV‐LRE	64.9 ± 4.4	65.9 ± 3.8	79.0 ± 3.2	72.0 ± 4.0	67.4 ± 5.0	67.1 ± 5.1
HRE	66.2 ± 4.6	70.4 ± 4.1	80.4 ± 2.7	70.8 ± 3.8	71.0 ± 4.0	68.8 ± 4.6

Note

Values are presented as Mean ± *SEM*.

^a^ Significant difference (*p* < .05) between pre‐exercise periods (i.e., Baseline and/or Pre‐EX) and Post‐EX 0 min.

^b^ Significant difference (*p* < .05) between pre‐exercise periods and Post‐EX 10 min.

^c^ Significant difference (*p* < .05) between pre‐exercise periods and Post‐EX 20 min.

^d^ Significant difference (*p* < .05) between pre‐exercise periods and Post‐EX 30 min.

^e^ Significant difference (*p* < .05) between Post‐EX 0 min and Post‐EX 10 min.

^f^ Significant difference (*p* < .05) between Post‐EX 0 min and Post‐EX 20 min.

^g^ Significant difference (*p* < .05) between Post‐EX 0 min and Post‐EX 30 min.

^h^ Significant difference (*p* < .05) between Post‐EX 10 min and Post‐EX 20 min.

^i^ Significant difference (*p* < .05) between Post‐EX 10 min and Post‐EX 30 min.

## 4 DISCUSSION

The primary finding of the present study was that the immediate and sustainable levels of postexercise IC improvements were similar between HV‐LRE and volume‐matched HRE. In a recent study, we determined that immediate postexercise IC improvement was greater with HRE than with LRE (Tsukamoto, Suga, et al., 2017), which was due to a difference in work volume. Therefore, the present findings suggest that work volume may play an important role in determining degrees of resistance exercise‐induced IC improvements.

We previously demonstrated that the degree of aerobic exercise‐induced IC improvements is associated with exercise volume (i.e., the product of exercise intensity and duration; Tsukamoto, Takenaka, et al., 2017). Nevertheless, in a previous study, we found that postexercise IC improvements were greater following aerobic moderate‐intensity exercise of 20‐min duration than following volume‐matched aerobic low‐intensity exercise of 40‐min duration (Tsukamoto, Takenaka, et al., 2017). Furthermore, in another study, we demonstrated that postexercise IC improvements could be more sustained following aerobic high‐intensity interval exercise than following volume‐matched aerobic moderate‐intensity continuous exercise (Tsukamoto et al., 2016a). Our previous findings suggest that exercise intensity alone, rather than exercise duration and volume, may be an important variable for determining the degree of aerobic exercise‐induced IC improvements. By contrast, the present findings demonstrated that work volume was the more important variable in determining the degree of resistance exercise‐induced IC improvements than exercise load and total repetitions. Therefore, a main variable of the postexercise IC improvements may be, at least partially, different between resistance and aerobic exercises.

To the best of our knowledge, only one study examined the effect of work volume on resistance exercise‐induced immediate improvements in IC. Engeroff et al. (2019) reported that among the three different intensities, according to volume‐matched whole‐body resistance exercise protocols (moderate‐intensity, 60% 1‐RM; high‐intensity, 75% 1‐RM; very high‐intensity, 90% 1‐RM), the use of moderate‐intensity led to the greatest immediate postexercise IC improvement. However, they did not have a fixed number of sets and duration of interset rest intervals among the three protocols. The differences in these variables among the resistance exercise protocols may influence the degree of postexercise IC improvements (Fortes et al., 2018), possibly by affecting some systems, including neuromuscular (Jenkins et al., 2015), metabolic (Kraemer et al., 1990), and perceptual systems (Woods, Bridge, Nelson, Risse, & Pincivero, 2004), for increasing neural activity in the brain. In the present study, we examined with the same number of sets and the same duration of interset rest intervals between HV‐LRE and HRE. Moreover, because whole‐body resistance exercise comprises multiple events, this exercise type may have additional effects on cognitive function due to the different movements being performed (Suzuki et al., 2012). By contrast, this confounding effect may be smaller for localized resistance exercise than for whole‐body resistance exercise because of one specific movement (i.e., only a knee extension). Furthermore, whole‐body resistance exercise involves long exercise duration, and therefore, this exercise type may require high aerobic demand throughout this exercise session, because long‐term whole‐body resistance exercise may increase aerobic capacity (e.g., VO_2 peak_; Kraemer et al., 1997). In the study by Engeroff et al. (2019), exercise duration of approximately 60 min was needed to complete the whole‐body resistance exercise. Similarly, most previous studies used whole‐body resistance exercise with exercise duration longer than 30 min (Alves et al., 2012; Brush et al., 2016; Chang et al., 2014). By contrast, exercise duration of the localized resistance exercise employed in the present study was shorter (i.e., 19 min for HV‐LRE and 17 min for HRE) than that of whole‐body resistance exercise employed in the previous studies (Alves et al., 2012; Brush et al., 2016; Chang et al., 2014; Engeroff et al., 2019); therefore, localized resistance exercise has lower aerobic demand during exercise session. Considering these findings, the factors involved in improving IC may be easier to understand in localized resistance exercise than in whole‐body resistance exercise. Therefore, the present findings can provide a clearer interpretation about the potential impact of work volume on resistance exercise‐induced IC improvements.

Little is known about the degree of postexercise IC improvements that can be sustained after resistance exercise. To the best of our knowledge, only a couple of studies have examined the sustainable effect of resistance exercise on IC (Brush et al., 2016; Johnson et al., 2016). Brush et al. (2016) reported that high‐intensity whole‐body resistance exercise‐induced IC improvements could not be sustained up to the 180‐min postexercise recovery period. Furthermore, Johnson et al. (2016) reported that moderate‐intensity whole‐body resistance exercise‐induced IC improvements could not be sustained up to the 30‐min postexercise recovery. The results of these previous studies suggest that the duration of IC improvements following resistance exercise may be less than 30 min, which may be a shorter duration compared with that after some aerobic exercise protocols obtained in our previous studies (Tanaka et al., 2018; Tsukamoto et al., 2016a, 2016b; Tsukamoto, Takenaka, et al., 2017). Nevertheless, in a study by Johnson et al., (2016) they recruited older individuals, and therefore, the sustainable effect of postexercise IC improvements may be limited in this population. In the present study, we determined that medium‐sized effects were observed between the ICs before exercise and 30 min after HV‐LRE and HRE, suggesting that the IC following both protocols could sustain moderate improvement throughout the 30‐min postexercise recovery period in young individuals. This duration of exercise‐induced IC improvements may be longer than that of low‐ or moderate‐intensity continuous exercise as seen in our previous studies (Tanaka et al., 2018; Tsukamoto et al., 2016a, 2017). These findings suggest that postexercise IC improvements induced by HV‐LRE and HRE may have a more sustainable effect than those induced by the traditional aerobic continuous exercise protocols. Taken together, the present study is the first to determine the sustainable effect of work volume on resistance exercise‐induced IC improvements.

The present study showed that the quadriceps femoris EMG activities were higher during HRE than during HV‐LRE. The increased muscle activity levels during resistance exercise may be associated with cerebral neural activation (Dai, Liu, Sahgal, Brown, & Yue, 2001; van Duinen, Renken, Maurits, & Zijdewind, 2008), which is probably the most prominent determinant of cognitive functions, including IC (Hyodo et al., 2016; Richeson et al., 2003). Thus, exercise‐induced increase in cerebral neural activity may be lower for HV‐LRE than for HRE. Nevertheless, EMG activities of the exercising muscles during HV‐LRE were longer than that during HRE because of the larger number of repetitions (i.e., 20 vs. 10 repetitions) per set for HV‐LRE than for HRE. Therefore, despite lower muscle activation, longer muscle activation of the exercising muscles may contribute, at least partially, to increasing cerebral neural activation and improving cognitive functions.

Systemic lactate is used in the human brain during exercise as an important energy substrate instead of glucose (van Hall et al., 2009). Our previous studies demonstrated that the degree of immediate and sustainable effects on aerobic exercise‐induced IC improvements is related to circulating lactate levels (Tanaka et al., 2018; Tsukamoto et al., 2016a, 2016b; Tsukamoto, Takenaka, et al., 2017), potentially due to increasing cerebral lactate metabolism (Hashimoto et al., 2018). In our previous study described above, the difference in postexercise IC improvements between volume‐matched aerobic exercise protocols (i.e., moderate‐intensity exercise with 20‐min duration vs. low‐intensity exercise with 40‐min duration) can be partially explained by the difference in exercise‐induced increase in blood lactate levels (3.3 ± 0.3 vs. 1.0 ± 0.1 mM at the end of exercise, respectively; Tsukamoto, Takenaka, et al., 2017). Such difference in exercise‐induced increase in blood lactate levels may also contribute to our understanding of a higher sustainable effect of postexercise IC improvements following high‐intensity interval exercise than that following moderate‐intensity continuous exercise (8.0 ± 1.0 vs. 2.7 ± 0.3 mM at the end of exercise, respectively; Tsukamoto et al., 2016a). In the present study, a significant main effect for condition and a trend toward significant interaction effect were observed with higher blood lactate levels following HV‐LRE than those following HRE. Therefore, compared with HRE, higher metabolic responses, such as increased blood lactate during HV‐LRE, may contribute to compensate its lower neuromuscular response (i.e., EMG activity).

Previous studies reported that perceptual responses, such as RPE and arousal, are associated with increases in cerebral neural activity and cognitive function (Byun et al., 2014; Fontes et al., 2015). In the present study, RPE during resistance exercise did not differ between HV‐LRE and HRE. Similarly, changes in FAS‐measured arousal following resistance exercise also did not differ between the two protocols. These present findings may also contribute to our understanding of similar postexercise IC improvements following LV‐LRE and HRE.

A major limitation of this study is that although this study recruited healthy young males, effective resistance exercise programs to improve cognitive function are generally more important for older individuals and patients with chronic diseases than for healthy young individuals. Further studies are required to examine the effect of the HV‐LRE on postexercise IC improvements in various populations.

## 5 CONCLUSION

This study demonstrated that even with a lower exercise load, HV‐LRE improves IC in a similar manner to volume‐matched HRE. Thus, we suggest that work volume is an important variable in determining the degree of IC improvements following resistance exercise. It is well known that HRE is a beneficial strategy for increasing muscle strength and size in various populations (American College of Sports Medicine, 2009; Williams et al., 2007). In addition to this, unique resistance exercise protocols that used lower intensity loads, such as the HV‐LRE and blood flow restriction exercise, have been determined to induce these muscle adaptations effectively (Mitchell et al., 2012; Takada et al., 2012; Takarada, Sato, & Ishii, 2002). In this regard, older individuals and patients with chronic disease often have difficulty with higher exercise loads due to declining health in cardiovascular and musculoskeletal systems. Therefore, in the clinical setting, information from the present findings may be useful in creating a safe and effective resistance training program for improving cognitive function in various populations.

## CONFLICT OF INTEREST

The authors declare no conflict of interest.

## AUTHOR CONTRIBUTIONS

K.T. and Tada. S. conceived and designed the experiment; K.T., Tada. S., Take. S., D.T., K.S., K.D., E.M., S.M., and H.T. performed experiments; K.T., Tada.S., Take.S., D.T., K.S., K.D., E.M., S.M., H.T., S.T., T.H., and T.I. interpreted results of experiments; K.T. and Tada. S. wrote the manuscript; H.T., S.T., T.H., and T.I. edited and revised the manuscript. All authors have read and approved the manuscript.
